# Vaccination with recombinant adenovirus expressing multi-stage antigens of *Toxoplasma gondii* by the mucosal route induces higher systemic cellular and local mucosal immune responses than with other vaccination routes

**DOI:** 10.1051/parasite/2017013

**Published:** 2017-04-03

**Authors:** Ting Wang, Huiquan Yin, Yan Li, Lingxiao Zhao, Xiahui Sun, Hua Cong

**Affiliations:** 1 Department of Human Parasitology, Shandong University, School of Medicine No. 44 Wenhuaxi Road Jinan Shandong 250012 P.R. China; 2 Shandong Xiehe University No. 6277 Jiqing Road Jinan Shandong 250107 P.R. China

**Keywords:** *Toxoplasma gondii*, Vaccine, Recombinant adenovirus, Mucosal vaccination

## Abstract

Toxoplasmosis caused by *Toxoplasma gondii*, an obligate intracellular protozoan, is a cause of congenital disease and abortion in humans and animals. Various vaccination strategies against toxoplasmosis in rodent models have been used in the past few decades; however, effective vaccines remain a challenge. A recombinant adenovirus vaccine expressing ubiquitin-conjugated multi-stage antigen segments (Ad-UMAS) derived from different life-cycle stages of *T. gondii* was constructed previously. Here, we compared the immune responses and protection effects in vaccination of mice with Ad-UMAS by five vaccination routes including intramuscular (i.m.), intravenous (i.v.), subcutaneous (s.c.), intraoral (i.o.), and intranasal (i.n.). Much higher levels of *T. gondii*-specific IgG and IgA antibodies were detected in the sera of the intraoral and intranasal vaccination groups on day 49 compared with controls (*p* < 0.05). The percentages of CD8^+^ T-cells in mice immunized intranasally and intraorally were larger than in mice immunized intramuscularly (*p* < 0.05). The highest level of IL-2 and IFN-*γ* was detected in the group with nasal immunization, and splenocyte proliferation activity was significantly enhanced in mice immunized via the oral and nasal routes. Furthermore, the higher survival rate (50%) and lower cyst numbers observed in the intraoral and intranasal groups all indicate that Ad-UMAS is far more effective in protecting mice against *T. gondii* infection via the mucosal route. Ad-UMAS could be an effective and safe mucosal candidate vaccine to protect animals and humans against *T. gondii* infection.

## Abstract (fr)

La toxoplasmose, causée par *Toxoplasma gondii*, un protozoaire intracellulaire obligatoire, est une cause de maladie congénitale et d’avortement chez l’homme et chez l’animal. Différentes stratégies de vaccination contre la toxoplasmose, chez les rongeurs, ont été employées au cours des dernières décennies, mais obtenir un vaccin efficace demeure un défi. Un vaccin d’adénovirus recombinant exprimant des segments d’antigènes multi-stades conjugués à l’ubiquitine (Ad-UMAS), dérivés de différents stades du cycle de vie de *T. gondii*, a été construit précédemment. Dans cette étude, nous avons comparé les réponses immunitaires et les effets de protection de la vaccination des souris avec Ad-UMAS par cinq voies de vaccination, y compris intramusculaire, intraveineuse, sous-cutanée, intra-orale et intranasale. Des taux beaucoup plus élevés d’anticorps IgG et IgA spécifiques de *T. gondii* ont été détectés dans le sérum des groupes de souris des vaccinations intra-orales et intranasale au jour 49, par rapport aux témoins (*p* < 0.05). Les pourcentages de lymphocytes CD8^+^ sont plus importants chez les souris immunisées par voie intranasale et intraorale que chez les souris immunisées par voie intramusculaire (*p* < 0.05). Le niveau le plus élevé d’IL-2 et d’IFN-y a été détecté dans le groupe d’immunisation nasale et l’activité de prolifération des splénocytes a été significativement augmentée chez les souris immunisées par voie orale et nasale. En outre, un taux de survie plus élevé (50 %) et plus petit nombre de kystes observés dans les groupes intra-oraux et intranasaux indiquent qu’Ad-UMAS est beaucoup plus efficace pour protéger les souris contre l’infection par *T. gondii* par voie muqueuse. Ad-UMAS pourrait être un candidat vaccin par voie muqueuse efficace et sûr pour protéger les animaux et les humains contre l’infection par *T. gondii*.

## Introduction

*Toxoplasma gondii* is a single-cell obligate intracellular protozoan with an infection rate of approximately 25%–30% in the worldwide population [6, 13, 29]. In pregnant women, this parasite may threaten health or even be fatal concerning congenitally infected fetuses [30]. It is also a major opportunistic infection in immunodeficient individuals, causing toxoplasmic encephalitis and retinochoroiditis [7, 17, 33]. As a result, an effective vaccine against *T. gondii* is urgently needed to prevent this disease.

Various vaccination strategies against toxoplasmosis in rodent models have been used in the last few decades. However, effective vaccines remain a challenge as tested vaccines have not been able to confer sterile immunity against infection [19]. Current research on *T. gondii* vaccines only focuses on antigens expressed in the tachyzoite stage, which only induce partial protective immunity [20, 28, 38, 44]. The development of a variety of epitope combinations from different stages of the life cycle, including tachyzoites, bradyzoites (in tissue cysts), and sporozoites (in oocysts), are likely to induce full protection [2, 3, 4, 43]. With the development of bioinformatics, epitope vaccines are considered a novel immunization approach to prevent *T. gondii* infection. In addition, an appropriate delivery system of vaccines could increase the transfection efficiency of immune cells.

Adenovirus, which can penetrate host cells delivering vaccine antigen to antigen-presenting cells (APCs) and elicit vigorous and sustained T-cell responses, is a promising *T. gondii* vaccine vector and can be used effectively to transport immunogens [27]. Recent studies have also found that recombinant canine adenovirus type-2 expressing TgROP16 and TgROP18 provides partial protection against acute *T. gondii* infection in mice, and indicate that adenovirus vectors may be potentially useful in the development of an effective vaccine against *T. gondii* infection [21, 22].

The mucosal immune system consisting of specialized epithelial cells can trigger both humoral and cell immune responses when antigens are administered with appropriate adjuvants or attenuated live vaccines via mucosal routes (oral, nasal, sublingual, ocular, genital, or rectal) [18, 23]. Furthermore, local mucosal immunization can lead to antigen-specific T- and B-cell responses not only in mucosal sites but also systemically [5, 24]. Hence, there is a greater need for effective vaccines that exert protective effects at mucosal surfaces, especially for intracellular parasites such as *T. gondii*.

In this research, we utilize a recombinant adenovirus vaccine expressing ubiquitin-conjugated multi-stage antigen segments (Ad-UMAS) derived from different life-cycle stages of *T. gondii* to evaluate the immune responses obtained with the different intramuscular vaccination routes described in our earlier study [41]. To further explore the optimal immune pathway for this recombinant adenovirus *T. gondii* vaccine, we plan to determine the immune responses and protection efficacy by vaccinating BALB/c mice via five routes (intramuscular (i.m.), intravenous (i.v.), subcutaneous (s.c.), intraoral (i.o.), and intranasal (i.n.)).

## Materials and methods

### Ethics approval

All the experimental procedures with animals used in the present study were granted prior approval by the Institutional Animal Care and Use Committee of Shandong University under Contract LL201602044. Humane endpoints are chosen to terminate the pain or distress of the experimental animals via euthanasia. Mice were monitored daily over 11 weeks for signs of toxoplasmosis including food and water intake difficulties, fatigue, severe ascites, and any test animals that showed signs of illness were euthanized immediately with CO_2_ gas.

### Parasites

Tissue cysts of the low-virulence Prugniaud (PRU) strain (type II) and tachyzoites of the high-virulence RH strain (type I) of *T. gondii,* which were a kindly provided by Professor Xingquan Zhu at Lanzhou Veterinary Research Institute, were propagated and harvested as described in our previous studies [41], and then used for the *in vivo* challenge of mice.

### Construction of the recombinant adenovirus vaccine

Ad-UMAS vaccine was constructed as previously described [40]. Briefly, Ad-UMAS was constructed using the AdMAX system (Hanbio, Shanghai, China). The ubiquitin-conjugated multi-stage antigen segments (UMAS) were cloned into a shuttle plasmid, pHBAd-MCMV-GFP. Ad-UMAS was generated by homologous recombination of pHBAd-MCMV-GFP-UMAS with pHBAd-BHG in HEK-293 cells. Ad-UMAS particles, with a titer of 10^11^ plaque-forming units (PFU)/mL, were purified by cesium chloride gradient centrifugation and then stored in storage buffer (10 mM Tris, 2 mM MgCl_2_, and 5% sucrose, pH 8.0) at −80 °C.

### Mice immunization

**Figure 1.**
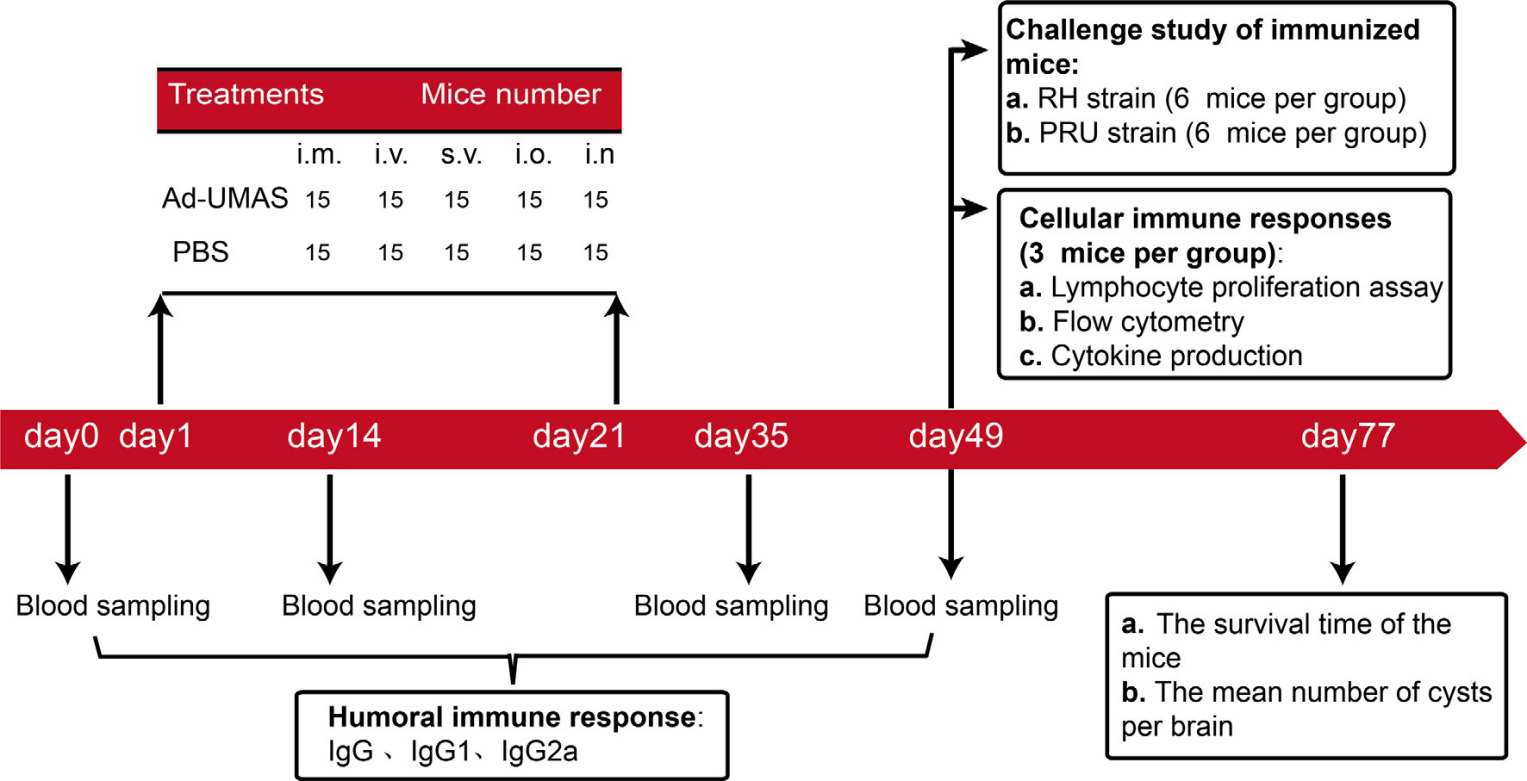
Study flowchart presenting groups of mice vaccinated with Ad-UMAS via different immunization routes.

Specific-pathogen-free female BALB/c mice aged 6–8 weeks were purchased from Shandong University Laboratory Animal Centre (Jinan, China). The animals for vaccination included intramuscular, intravenous, subcutaneous, intraoral, and intranasal immunization groups (15 mice per group). For the intramuscular group, the mice were injected 100 μL (3 × 10^8^ PFU) Ad-UMAS in the quadriceps muscle. For the intravenous group, the mice were injected 50 μL (3 × 10^8^ PFU) Ad-UMAS in the caudal vein. The subcutaneous and oral groups were administered 200 μL (3 × 10^8^ PFU) Ad-UMAS by subcutaneous injection and intragastric administration. For intranasal vaccinations, mice were anesthetized with 3% isoflurane in oxygen and were given a nasal drip of 10 μL (3 × 10^8^ PFU) Ad-UMAS with the head canted 45° for 10 min. Each group also had 15 mice that were treated with phosphate-buffered saline (PBS) as the control. Mice were vaccinated twice at 3-week intervals. Table 1 summarizes the treatments given to the mice. Figure 1 shows the study flowchart for vaccinated mice.

**Table 1.** Summary of treatments in BALB/c mice.

Vaccination pathway^a^	Treatments^b^	Mice number^c^
Intramuscular	100 μL PBS	15
	100 μL (3 × 10^8^ PFU) Ad-UMAS	15
Intravenous	50 μL PBS	15
	50 μL (3 × 10^8^ PFU) Ad-UMAS	15
Subcutaneous	200 μL PBS	15
	200 μL (3 × 10^8^ PFU) Ad-UMAS	15
Intraoral	200 μL PBS	15
	200 μL (3 × 10^8^ PFU) Ad-UMAS	15
Intranasal	20 μL PBS	15
	20 μL (3 × 10^8^ PFU) Ad-UMAS	15

a The mice were randomly divided into intramuscular, intravenous, subcutaneous, intraoral, and intranasal immunization groups (15 mice per group).

b For the intramuscular group, the mice were injected 100 μL (3 × 10^8^ PFU) Ad-UMAS vaccine in the quadriceps muscle. For the intravenous group, the mice were injected 50 μL (3 × 10^8^ PFU) Ad-UMAS vaccine in the caudal vein. The subcutaneous and oral groups were each administered 200 μL (3 × 10^8^ PFU) Ad-UMAS vaccine. For the nasal group, each mouse was given a nasal drip of 20 μL (3 × 10^8^ PFU) Ad-UMAS vaccine.

c Each group also had 15 mice which were treated with PBS as negative control. Mice were vaccinated twice at 3-week intervals.

### Measurement of humoral response

Serum samples were collected by retro-orbital bleeding on days 0, 14, 35, and 49. Standard ELISAs were used to determine the levels of *T. gondii*-specific antibodies, IgG, IgG1, IgG2a, and IgA, in the serum samples from the inoculated mice. This was done with some changes to the method previously described [3, 5]. Briefly, a flat-bottom 96-well plate was pre-coated with the UMAS peptide pool at a concentration of 10 μg/mL in a 50 mM carbonate-bicarbonate buffer (pH 9.6) overnight at 4 °C. The mouse sera were diluted in PBS (1:100) and incubated at 37 °C for 1 h. After washing the plates, bound antibodies were then reacted with horseradish peroxidase (HRP)-conjugated goat anti-mouse IgG, IgG1, IgG2a, or IgA (Sigma-Aldrich, USA) at 37 °C for 1 h. Peroxidase activity was revealed by 3, 3′, 5, 5′-tetramethylbenzidine (TMB, 10 mg/mL) and stopped by adding 50 μL of 2 M H_2_SO_4_. The optical density (OD) at 450 nm was measured by a Thermo Scientific Multiskan FC Microplate Photometer (Thermo Scientific, USA).

### Lymphocyte proliferation assay

Spleens were removed from three immunized mice per group four weeks after the last immunization. Single-cell splenocytes were harvested following the method described in a previous study [5, 42]. Isolated splenocytes were plated in 96-well plates, at a density of 1 × 10^6^ per well, in 100 μL RPMI-1640 medium (Sigma-Aldrich, USA) supplemented with 10% fetal calf serum and cultured with Concanavalin A (ConA, 2 μg/mL, Sigma-Aldrich) or the UMAS peptide pool (10 μg/mL). Cell proliferative activity was measured according to the manufacturer’s instructions on a Dojindo Cell Counting Kit-8 (Dojindo, Japan). The results were expressed as absorbance at 450 nm.

### Cytokine production

The level of cytokine production was determined using splenocytes from three mice per group 4 weeks after the last immunization. Commercial ELISA Kits (R&D Systems, USA) were used following the manufacturer’s instructions to assay IL-2 at 24 h, IL-10 at 72 h, and IFN-*γ* at 96 h in culture supernatants obtained as previously described [40]. This was done by collecting the cell supernatant of three wells from the 96-well plates and centrifuging to discard cell debris. The supernatant was tested for cytokines. The detection was replicated three times for each spleen.

### Cell surface staining of murine splenic lymphocytes

Cells were stained with fluorescein isothiocyanate (FITC)-labeled anti-mouse CD8^+^ monoclonal antibody and phycoerythrin (PE)-labeled anti-mouse CD4^+^ monoclonal antibody; T lymphocyte subsets were measured by a Beckman Coulter FC500 Flow Cytometer (Beckman Coulter, USA).

### Challenge study of immunized mice

Four weeks after the last immunization, mice were challenged with the type 1 or type 2 parasites to evaluate the protective effect. Six mice per group were infected intraperitoneally with 1 × 10^3^ tachyzoites of *T. gondii* RH strain (type 1 parasite) after the last immunization, and the survival time of the mice was observed and recorded.

Another six immunized mice per group were infected via the gastric route with 20 cysts of the *T. gondii* PRU (type 2 parasite) strain. The challenged mice were sacrificed 4 weeks later and the brains were removed and homogenized in 1 mL PBS. The mean number of cysts per brain was determined by counting three samples of 10 μL aliquots from each homogenized brain under an optical microscope [20].

### Statistical analysis

Statistical significances between the groups were calculated by one-way analysis of variance (ANOVA) using SPSS 19.0 software. The survival rate was compared by the Kaplan-Meier method. A *p*-value of less than 0.05 (*p* < 0.05) was considered to be significant.

## Results

### Humoral IgG antibody was induced via all immunization routes

**Figure 2.**
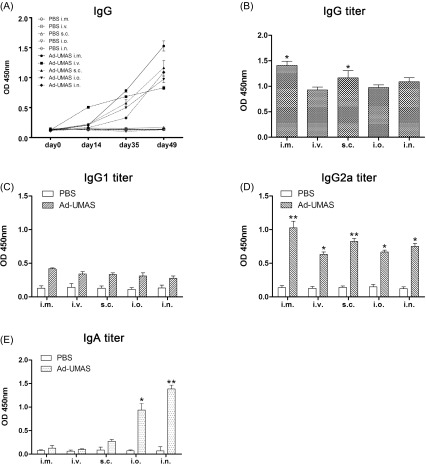
Systemic humoral immune response and local mucosal immune response induced by Ad-UMAS via different immunization routes. (A) IgG antibodies detected in murine serum collected on days 0, 14, 35, and 49. (B) IgG, (C) IgG1, (D) IgG2a, and (E) IgA titers were detected using sera from 4 weeks after the last vaccination. The results are expressed as the mean of the OD450 ± *SD* and are representative of three experiments. Asterisks mark the significant difference: *p < 0.05; **p < 0.01. Each bar represents the mean OD (± *SD*, *n* = 15).

To assess the systemic humoral immune responses obtained with the five immunization routes of Ad-UMAS, we collected sera at different times after the immunization, and anti-*T. gondii* IgG and IgG subtypes were detected by ELISA. As shown in Figure 2A, the levels of antibody titers all increased in the five Ad-UMAS immunization routes. Compared to the control, levels of *T. gondii*-specific IgG antibodies increased dramatically in the sera of all vaccination mice from day 14 to day 35 and achieved the highest on day 49 (*p* < 0.05). Among different immunization routes, the intramuscular group achieved the highest titer of IgG antibodies, then the subcutaneous, nasal, and oral groups, in decreasing order (Fig. 2B). In the intravenous vaccination group, the titer of IgG increased rapidly at 2 weeks and gradually more slowly thereafter, making the final titer relatively low.

Further evaluation of serum IgG subtype antibody revealed that *T. gondii*-specific IgG1 and IgG2a antibody levels were both significantly increased in five Ad-UMAS vaccination groups as compared with the control (*p* < 0.05), while their differences were substantial in IgG2a but not in IgG1. The values of IgG2a antibody were relatively higher in the intramuscular and subcutaneous vaccination groups than with other vaccination routes (Figs. 2C and 2D).

### Higher mucosal immune responses were induced by intraoral and intranasal vaccination of Ad-UMAS

To evaluate the ability of Ad-UMAS to induce local mucosal immune responses via the five pathways, mucosal IgA antibody response was detected in the collected mouse sera.

As shown in Figure 2E, vaccination in the intraoral and intranasal groups elicited strong *T. gondii*-specific IgA antibody levels that were significantly higher than other vaccination routes (*p* < 0.05) (Fig. 2E). However, there were no statistically significant differences of IgA titer in the intramuscular, intravenous, and subcutaneous vaccination groups compared to control groups (*p* > 0.05).

### Robust cellular immune responses were induced via intranasal vaccination

Cellular immune responses were first evaluated by analysis of the T-cell subset. The CD4^+^ and CD8^+^ T lymphocyte subsets in immunized mice were assayed by flow cytometry. Table 2 shows the percentage of CD4^+^ and CD8^+^ T-cells, in comparison with control groups; the percentage of CD8^+^ T-cells in all vaccination groups with Ad-UMAS increased significantly (*p* < 0.05). The percentages of CD8^+^ T-cells in mice immunized intranasally (36.5 ± 1.8%), intraorally (29.7 ± 0.2%), and intramuscularly (27.6 ± 1.3%) were higher than in mice immunized by the subcutaneous and intranasal routes (*p* < 0.05).

**Table 2.** CD4^+^ and CD8^+^ subtypes of T-cells from immunized mice measured using flow cytometry.

Immunization routes^a^	Immunization regimen	Percentage of positive stained cells (%: mean ± *SD n* = 3)^b^		CD4^+^/CD8^+^
		CD4^+^	CD8^+^	
Intramuscular	PBS	21.6 ± 0.9	10.7 ± 0.6	2.02
	Ad-UMAS	35.7 ± 0.8	27.6 ± 1.3	1.29
Intravenous	PBS	20.3 ± 1.2	9.6 ± 0.4	2.11
	Ad-UMAS	25.6 ± 1.0	18.9 ± 1.4	1.35
Subcutaneous	PBS	19.1 ± 0.7	9.3 ± 0.6	2.05
	Ad-UMAS	28.5 ± 0.4	22.6 ± 1.2	1.26
Intraoral	PBS	21.4 ± 0.5	11.2 ± 0.8	1.91
	Ad-UMAS	37.4 ± 0.7*	29.7 ± 0.2*	1.26*
Intranasal	PBS	20.9 ± 1.3	10.1 ± 0.8	2.07
	Ad-UMAS	40.6 ± 1.1**	36.5 ± 1.8**	1.11**

a Mice were immunized by five immunization routes, intramuscular, intravenous, subcutaneous, intraoral, or intranasal, on day 0 and day 21 with different immunization regimens.

b The splenocyte culture supernatants taken from mice (*n* = 3, each group) 2 weeks after the last immunization were stained with FITC-labeled anti-mouse CD8^+^ monoclonal antibody and PE-labeled anti-mouse CD4^+^ monoclonal antibody; T lymphocyte subsets were measured using flow cytometry. Asterisks mark the significant difference:

* *p* < 0.05;

** *p* < 0.01.

Each bar represents the mean OD (± *SD*).

The cell-mediated immunity induced in the immunized mice was further evaluated by measuring the amount of cytokines IL-2, IL-10, and IFN-γ. Table 3 shows the values of IL-2, IFN-γ, and IL-10 in all vaccination groups. The highest levels of IL-2 and IFN-γ were detected in the nasal immunization group at 638.7 ± 17.6 pg/mL and 1429.8 ± 37.6 pg/mL, respectively. Furthermore, the lymphocyte proliferation ability was also higher in the mice inoculated nasally with Ad-UMAS than the controls (*p* < 0.05). However, there were no statistically significant differences in the amount of IL-10 between all the immunization group and control groups (*p* > 0.05) (Table 3).

**Table 3.** Cytokine production and cell proliferative assay in the splenocyte cultures obtained from immunized mice.

Immunization routes^a^	Immunization regimen	Cytokine production (pg/mL)^b^			Stimulation index^c^
		IL-2	IL-10	IFN-γ	
Intramuscular	PBS	78 ± 8.5	51 ± 9.7	103 ± 9.7	0.37
	Ad-UMAS	454.2 ± 15.1	48.7 ± 5.1	1085.6 ± 25.1	1.52
Intravenous	PBS	56 ± 9.3	43 ± 8.1	92 ± 6.4	0.25
	Ad-UMAS	320.5 ± 9.6	55.5 ± 3.6	920.1 ± 19.6	0.94
Subcutaneous	PBS	86 ± 10.4	64 ± 4.6	83 ± 12.7	0.31
	Ad-UMAS	425.4 ± 13.7	54.3 ± 6.7	989.1 ± 43.7	1.04
Intraoral	PBS	74 ± 7.8	57 ± 8.3	86 ± 7.8	0.62
	Ad-UMAS	527.2 ± 8.1*	58.2 ± 5.1	1204.2 ± 28.1*	2.13*
Intranasal	PBS	63 ± 11.7	62 ± 6.7	98 ± 10.5	0.58
	Ad-UMAS	638.7 ± 17.6**	42.1 ± 2.6	1429.8 ± 37.6**	2.93**

a Mice were immunized by five immunization routes: intramuscular, intravenous, subcutaneous, intraoral, and intranasal on day 0 and day 21 with different immunization regimens.

b The splenocyte culture supernatants taken from mice (*n* = 3, each group) 2 weeks after the last immunization were examined for cytokine production by ELISA obtained at 24 h for IL-2, at 72 h for IL-10, and 96 h for IFN-γ.

c The results of proliferation assays are expressed as the stimulation index, calculated as the ratio between the mean counts per minute (cpm) for triplicate stimulated cultures and the mean counts per min for triplicate unstimulated cultures. Asterisks mark the significant difference:

* *p* < 0.05;

** *p* < 0.01.

Each bar represents the mean OD (± *SD*).

### Protective efficacy against the type I and type II parasites

**Figure 3.**
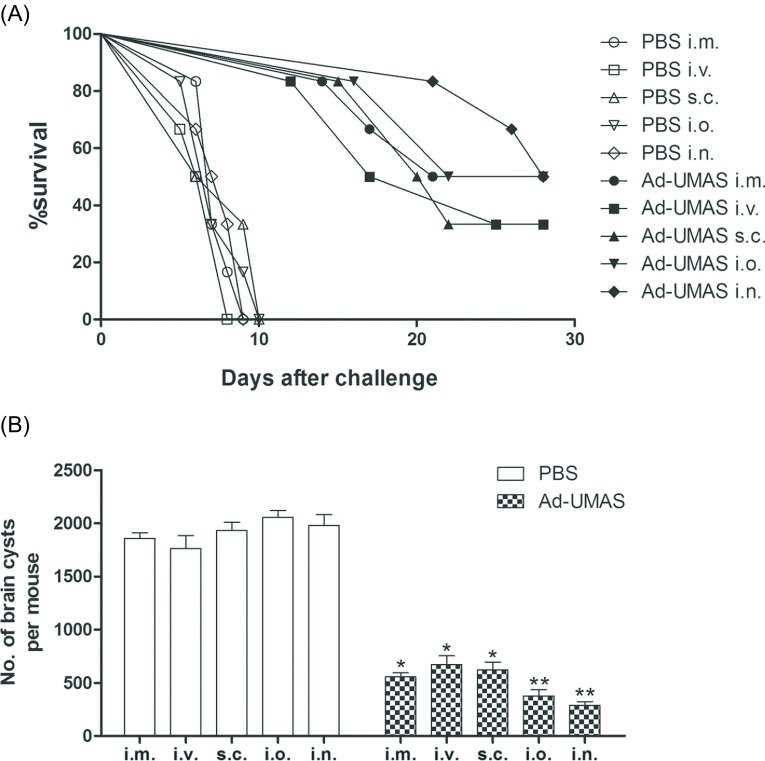
Evaluation of the protective ability against *T. gondii* infection*.* (A) Six immunized mice per group were infected intraperitoneally with 1 × 10^3^ tachyzoites of *T. gondii* RH strain 4 weeks after the last immunization. Survival was monitored daily for 28 days after challenge. (B) Another six immunized mice per group were infected intragastrically with 20 cysts of *T. gondii* PRU strain and the cyst burden in the brain of vaccinated mice was counted 4 weeks later. The mean number of cysts in every mouse group was based on each mouse brain cyst in the group. Asterisks mark the significant difference: *p < 0.05; **p < 0.05. Each bar represents the mean OD (± *SD*).

To evaluate the protective immunity, six mice of each group were given an intraperitoneal injection of 1 × 10^3^ tachyzoites of *T. gondii* RH strain at 4 weeks after vaccination. The survival rates of mice immunized via different routes are shown in Figure 3. The mice immunized with Ad-UMAS via i.m., i.o., and i.n. showed an almost 50% survival rate and lower survival rates of 40% were found in the i.v. and s.c. immunization groups 28 days after challenge. All the control mice died within 8 days.

The level of protective effect against chronic infection was evaluated by counting cysts in the brains of mice challenged with 20 cysts of the PRU strain of *T. gondii* (Fig. 3). An obvious reduction in the burden of brain cyst was detected in all the immunized mice compared with the control mice (*p* < 0.05). The brain cyst numbers in the oral and nasal immunization groups were significantly reduced to 459 ± 34 and 398 ± 61, respectively, which is much lower than the other immunization groups with injection in muscle (569 ± 23), via the venous route (892 ± 51), and via the subcutaneous route (675 ± 65) (*p* < 0.05).

## Discussion

The antigen delivery pathway is a key parameter for the induction of protective immune responses by vaccines against intracellular pathogens [10]. As a novel inoculation, there is general agreement that effective mucosal vaccines (oral, nasal, sublingual, and genital tract vaccines) could dramatically stimulate protective immune responses not only against mucosal infections but also against HIV, *Mycobacterium tuberculosis*, and many other pathogens [8, 37]. In this study, we compared the capability of Ad-UMAS to induce systemic humoral and cellular immune responses, and local mucosal immune responses via five vaccination pathways (i.m., i.v., s.c., i.o., and i.n.).

Mucosal vaccines are advantageous compared with systemic vaccines from a production and regulatory perspective [18, 23]. Previous studies have revealed that a Middle East respiratory syndrome (MERS) vaccine greatly enhances local mucosal immune responses in immunized BALB/c mice with the intranasal inoculation method [24]. Ideal attenuated vaccines could be engineered to have a limited capacity for replication and to deliver an effective antigen, while avoiding unwanted inflammatory responses [35, 39, 41]. Recombinant adenoviruses are some of the most intensively investigated vaccine carriers in many pathogenic infections, such as Ebola virus, HIV, and the malaria parasite [1, 11, 16, 32]. Several clinical trials have shown that vaccines based on adenoviruses could elicit vigorous and sustained T-cell responses, with the advantage of safety and high immunogenicity [25].

Our results showed that the Ad-UMAS vaccine could effectively improve the humoral response intramuscular pathway. Systemic humoral IgG antibody responses induced by the intraoral and intranasal vaccination pathway were comparable to those induced by the other pathway in the collected mouse sera. Interestingly, we found that IgG antibody in the intravenous vaccination group increased very sharply at first but slowly later on, even after the boost immunization. It should be considered that antigens that are transmitted to the bloodstream directly can induce strong immune responses in the early stage, but can also cause immune tolerance, explaining why antibody levels increase slowly in the following time [25].

Moreover, evaluation of serum IgG subtype antibody responses revealed that all vaccination pathways induced a relatively higher level of Th1-associated IgG2a than Th2-associated IgG1 in this study. It should be noted that Ad-UMAS induced a slightly biased Th1-associated antibody response. It is known that production of IgG subtype is driven by cytokines secreted during cellular immune responses. Therefore, the presence of IgG1 and IgG2a indirectly suggests that the vaccination protocol also promoted activation of a cell-mediated response [12, 31]. These data suggest that Ad-UMAS immunization by the mucosal vaccination route was capable of eliciting similar systemic humoral antibody responses compared with those elicited by the intramuscular pathway in vaccinated mice.

Mucosal vaccines against *Toxoplasma* infection require the induction of protective responses at mucosal surfaces and also the induction of systemic protective immune responses in the systemic compartment [18]. As the first line of defense in protecting mucous membranes, specific SIgA plays a protective role against many pathogens. Therefore, vaccines capable of inducing strong local mucosal immunity represented by secretory IgA would be helpful in preventing *T. gondii* infection [9]. Our data indicated that *T. gondii*-specific IgA antibody responses in vaccinated mouse sera were elicited by i.n. and i.o vaccination of Ad-UMAS, which were significantly higher than those induced by other vaccination routes, confirming the ability of Ad-UMAS to induce strong mucosal immunity via the mucosal route.

The memory T-cell consists of CD4^+^ and CD8^+^ T-cells, which play a vital role in the establishment of protective immunity in hosts [31]. CD8^+^ T-cells can control the spreading and development of *T. gondii* infection in synergy with CD4^+^ T-cells [12]. In our study, CD4^+^ and CD8^+^ T lymphocyte subsets from immunized mice were assayed by flow cytometry. There was a marked increase in the percentage of CD8^+^ T-cells in mice immunized with the Ad-UMAS vaccine via mucosal routes. Cytokines play a critical role in the activities of Th cells. As we know, Th1 cells are responsible for limiting tissue extension of the parasite through the production of IL-2 and IFN-γ [15]. Our results show that in contrast with the blank control, high levels of IL-2 and IFN-γ were induced in mice immunization with Ad-UMAS especially treated intraorally and intranasally. However, there is no difference in IL-10 production between vaccinated and control groups. Additionally, splenocyte proliferation activity was significantly enhanced in mice immunized via oral and nasal routes. These results clearly suggest that mucosal vaccination with Ad-UMAS can significantly augment Th1-mediated cellular immune responses in which CD8^+^ T lymphocytes are considered as major effectors responsible for controlling parasite infection and secreting IFN-γ during the cellular response against toxoplasmosis.

In our study, a highly virulent RH strain and a mild virulent PRU strain of *T. gondii* were used for the challenge study. With the RH strain, it is easy to induce acute infection in mice through intraperitoneal injection with tachyzoites. The PRU strain is more likely to induce chronic infection in mice following intragastric administration with cysts [36]. In order to simulate acute and chronic infection in the host, mice were infected with the RH strain and PRU strain by different challenge routes. When challenged with lethal doses of *T. gondii* (1 × 10^3^), all five groups immunized with Ad-UMAS had a prolonged survival time as compared with control mice. In particular, mice immunized intranasally and intraorally showed higher survival rates than with the other immunization routes. Since protection via vaccination was not 100% because the strain of the parasite has high mortality and the dose of RH parasites for immunized mice was larger, the optimal challenge dose should be determined in the future. Furthermore, the higher mucosal immune responses that were induced by intraoral and intranasal vaccination of Ad-UMAS may explain the significant reduction in the cyst burden in the mice immunized with Ad-UMAS intraorally and intranasally, which exhibited reductions of 75.3% and 78.6% of brain cysts, respectively.

Earlier studies [14, 26, 34] have determined mortality, cytokine production, and parasite burden after challenge infection in susceptible and resistant strains of mice with the same or similar doses of *T. gondii* parasites. These studies found that different inbred strains of mice have markedly different susceptibility to *T. gondii* infection. These susceptibility differences may be due to differences in the virulence of the parasite itself or differences in the genetic make-up of the mice in terms of their immune response. In our study, only one inbred strain of mice was used for the experiment. It is therefore necessary to assess the different immunizations in different strains of mice in future research.

In summary, mucosal immunization with recombinant adenovirus vaccine expressing ubiquitin-conjugated multi-stage antigen segments (Ad-UMAS) is a potential vaccination route to elicit protective immune responses. Compared to other immunization routes, oral and nasal immunizations with Ad-UMAS elicit both robust systemic and mucosal immune responses, accompanied by a significant increase in survival rates after challenge with highly virulent parasites and a dramatic reduction in brain cyst burdens in vaccinated mice after challenge with mild strains of *T. gondii*. Therefore, the results of this study should contribute to the development of an effective and safe mucosal vaccine for preventing *T. gondii* infection.

## Conflict of interest

The authors declare that they have no competing interests.
